# Ethno-ornithology and conservation of wild birds in the semi-arid Caatinga of northeastern Brazil

**DOI:** 10.1186/1746-4269-9-14

**Published:** 2013-02-27

**Authors:** Rômulo Romeu Nóbrega Alves, Railson Cidennys Lourenço Leite, Wedson Medeiros Silva Souto, Dandara M M Bezerra, Alan Loures-Ribeiro

**Affiliations:** 1 Departamento de Biologia, Universidade Estadual da Paraíba, Av. das Baraúnas, 351/Campus Universitário, Bodocongó, 58109-753, Campina Grande-PB, Brazil; 2 Programa de Pós-Graduação em Ciências Biológicas (Zoologia), Departamento de Sistemática e Ecologia da Universidade Federal da Paraíba, 58051-900, João Pessoa, PB, Brazil

## Abstract

The utilization of birds as pets has been recognized as one of the principal threats to global avifauna. Most of the information about the use and sale of birds as pets has been limited to areas of high biodiversity and whose impacts of anthropic actions have been widely broadcast internationally, for example for the Amazon Forest and forest remnants of Southeast Asia. The Caatinga predominates in the semi-arid region of Brazil, and is one of the semi-arid biomes with the greatest biological diversity in the world, where 511 species of birds exist. Many of these birds are used as pets, a common practice in the region, which has important conservationist implications but has been little studied. Therefore, the aim of the present study was to detail aspects of the use of birds as pets in a locality in the semi-arid region of Northeast Brazil. Information on the use of avifauna was obtained through interviews and visits to the homes of 78 wild bird keepers. A total of 41 species of birds were recorded, mostly of the families Emberizidae (n = 9 species), Columbidae (n = 7 species), Icteridae (n = 6 species) and Psittacidae (n = 3 species). The birds that were most often recorded were *Paroaria dominicana* (n = 79 especimens), *Sporophila albogularis* (n = 67), *Aratinga cactorum* (n = 49), *Sporophila lineola* (n = 36), *Sicalis flaveola* (n = 29) and *Sporophila nigricollis* (n = 27). The use of wild birds in the area studied, as an example of what occurs in other places in the semi-arid Northeast, demonstrates that such activities persist in the region, in spite of being illegal, and have been happening in clandestine or semi-clandestine manner. No statistically significant correlation were found between socioeconomic factors and keeping birds as pets reflects the cultural importance of this practice of rearing wild birds for pets in the region, which is widespread among the local population, independent of socioeconomic factors. Obviously, human pressure on the avifauna exploited has ecological implications and makes it clear that conservationist measures should consider the cultural, economic and social aspects of these practices. These measures should be carried out by both directly combating the illegal traffic of animals and promoting educational campaigns aimed at all the players involved, from the collectors up to the consumer and wild bird keepers.

## Introduction

There is no doubt that many human activities have reflected in important threats to the avifauna, especially tropical birds. About 95% of threatened birds worldwide suffer severe impacts as a result of habitat loss, whereas 71% are associated with various forms of uses by humans [1]. As a consequence, the population decline of many bird species has been influenced directly or indirectly by anthropic actions. Birds have been utilized for millenia for numerous purposes, from traditional use as food to exploitation of their parts as adornments and decorative accessories, and even for traditional medicine [2-14].

Unfortunately, there are a number of factors that negatively impact avifaunal structures, principally involving the loss and degradation of habitats and over-exploitation of bird populations [15-22] but also including the introduction of exotic species, pollution, natural disasters and road-kills [23-26]. Both the hunting and capture of bird species have been shown to affect their natural populations – with immediate and evident ecological implications [9,15,25,27-29].

The maintenance of wild birds in captivity, a widely spread practice among different cultures tracing back thousands years, is pointed out as one of the reasons for the decline in population of many species [28,30-34]. Of all the known birds in the world, 3,649 species (37% of the known total) are widely utilized as pets, such that the exploitation of birds as pets is undoubtedly the main pressure for the direct use of this taxon. This type of exploitation of birds, together with hunting for food, traditional remedies or ornaments, extends the number of 4,561 species of birds directly used by humans, i.e., 46% of about 10,000 species of known birds [35].

Brazil is home to one of richest faunas of birds in the world with 1,832 species [28,36,37]. Such numbers represent about 57% of the total species of birds recorded in South America [23]. More than 10% of this number are endemic to Brazil, making this one of the most important countries for investments in conservation [38]. However, in the same way that Brazil excels in richness of birds, the country also has the highest number of species threatened in the Neotropics [32]. In total, 189 species of birds are present on the global list of threatened species [39], and 160 on the national list [40]. This worrisome scenario follows the same general panorama of other areas in the tropics where massive habitat loss and indiscriminate utilization of birds has led many species to extinction [1].

In Brazil, the practice of keeping birds in cages is common in both rural and urban areas [10,23,28,41,42]. From large cities to small towns, caged birds can be found in commercial and residential establishments. Birds, however, are often captured in the natural environment and rarely obtained from legal venders [28,43]. In various locations, the practice of keeping birds in cages is so culturally important that people even use ornamental cages or even cages containing imitation birds [28,43,44].

In the semi-arid region of Brazil, birds are utilized for different purposes and are of great social, economic and cultural importance. In the Caatinga, there are 511 species of birds [45,46], some of which are often utilized by the local people as food (meat, eggs and bones), remedies (traditional medicine), and ornamental items (eggs and feathers), besides being also used for pleasure, companionship and ornamentation (canaries, pets) [28]. It is very common in the region to rear birds in cages [43,47]. Unfortunately some used birds are on the lists of threatened species [44,48].

In this scope, the importance of ethno-ornithological studies is clearly evident, since to make the sustainable use of avifauna possible, it is necessary to understand its interaction with the local inhabitants, its different forms of use and which species are more often utilized [28,49]. Besides, investigations on the use of birds contribute to ways in which these animals are duly valued not only from an ecological but also economic and social points of view [19,28]. Despite their value as a source of protein, the high frequency of game birds targeted is primarily related to their use as pets [5,9,10,28,50,51]. This value represents a strong stimulatory factor for the illegal trade of birds in the Caatinga. Various cities in the interior of northeast Brazil have public markets and open fairs where birds and other wild animals are sold [10,44].

In view of this scenario, the establishment of efficient conservation measures requires an understanding of the cultural social context associated with the use of wild birds in the Caatinga. Such information can be obtained through ethno-ornithological studies, which are still scarce in Brazil. Only 11 studies with this focus have been conducted specifically in the Caatinga [5,9,28,44,50-56], of which only one [28] presents quantitative data on the use of birds as pets. Therefore, the necessity for more research on this subject is clearly evident, because only in this way will we be able to resolve such questions as: Does the richness of bird species raised as pets in semi-arid northeastern Brazil vary with locality? Although a large number of species are utilized, are some more commonly kept as pets? Is the choice of the species raised in captivity related to the species’ conservation status? Or, are threatened species rarely kept in captivity, reflecting their scarcity in the environment? What is the influence of the socioeconomic aspects on this activity? Since answers to these questions should be useful in helping to contribute to our knowledge of the practice of keeping birds and their implications in the semi-arid region of northeastern Brazil, the present work was designed to learn about the species of wild birds that are raised as pets in the semi-arid region of the state of Paraíba, Northeast Brazil, and to evaluate conservationist aspects.

## Methods

### Study area

**Figure 1:**
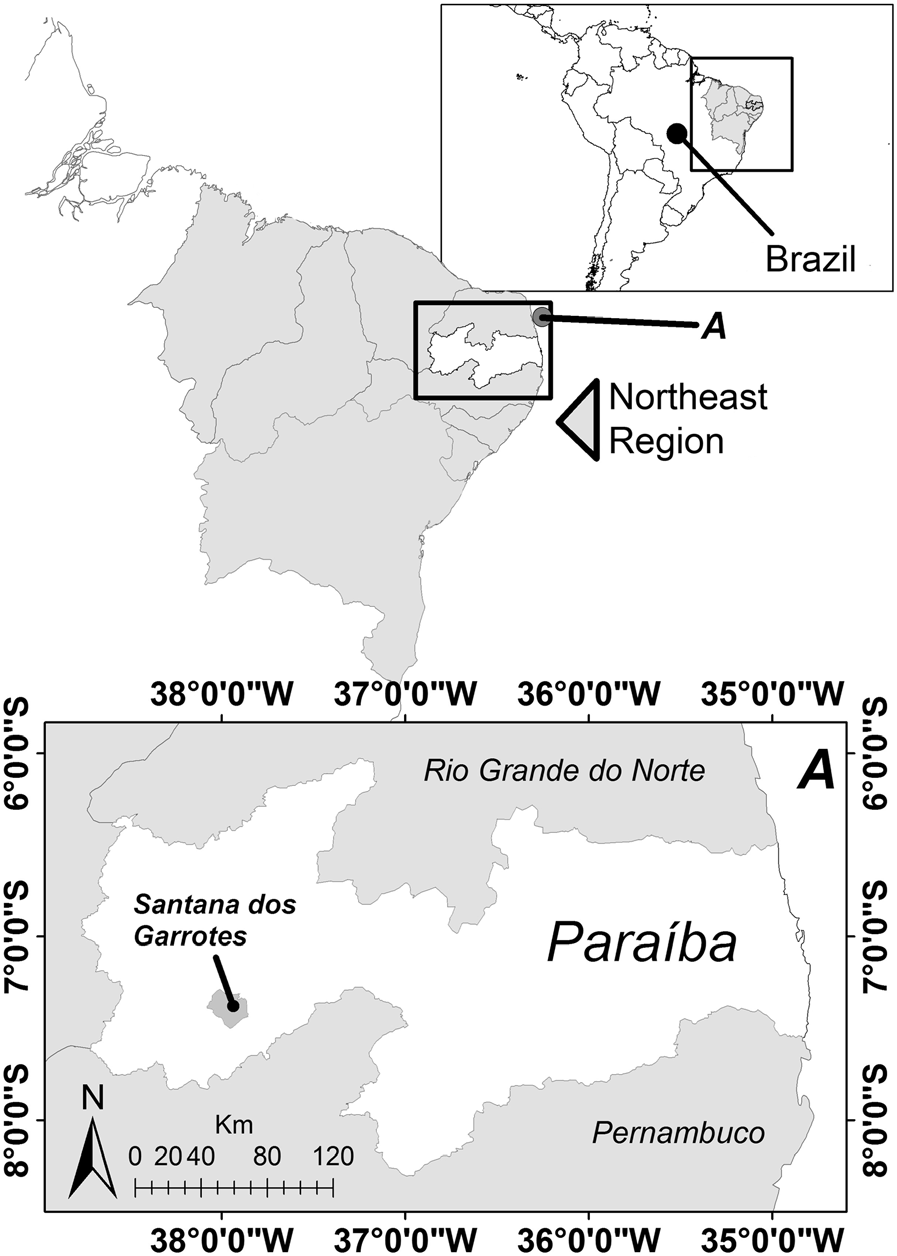
Location of the city of Santana dos Garrotes (Paraíba State, Northeast Brazil), where the study was conducted.

The study was conducted in the municipality of Santana dos Garrotes (07° 23′ 02″ S and 37° 59′ 09″ W) located in the Mesoregion of the Paraíba, Northeast Brazil (Figure 1). Santana dos Garrotes has an area of 353.813 km^2^ and a total population of 7, 266 inhabitants [57]. The municipality is within the so-called “Polígono das Secas” or Drought Polygon constituting a climate of the hot and dry semi-arid type, according to the Köppen classification. The temperatures are high during the day, easing at night, with annual variations of 23 to 30°C, with occasional higher peaks mainly in the dry season. Rainfall, besides being low, is irregular with annual means of 726.6 mm/year. In general, it is characterized by the presence of only two seasons: a short rainy season of 3 to 5 months, referred to as “winter” by local inhabitants, which occurs in the first half of the year, and a long dry season called “summer,” which lasts 7 to 9 months [58]. The vegetation is small-sized, typical of the xerophytic Caatinga, featuring cacti, shrubs and small- to medium-sized trees [59]. Agriculture and commerce are the main economic activities of the municipality. Demographics of the interviewees are summarized in Table 1.

**Table 1:** Information on educational attainment, age, income, and gender of interviewees (n = 78)

	
**Gender**	
Male	55 (70.5%)
Female	23 (29.4%)
**Age**	
Less than 30 years old	31 (39.7%)
30–39	12 (15.4%)
40–49	13 (16.6%)
50–59	9 (11.5%)
60 or older	13 (16.6%)
**Educational attainment**	
Illiterate	28 (35.9%)
Primary level incomplete	21 (26.7%)
Primary level complete	1 (1.3%)
Secondary level incomplete	16 (20.5%)
Finished high school	9 (11.5%)
Higher education incomplete or complete	3 (3.8%)
Monthly income^*^	
Less than US$ 150	30 (38.4%)
Between US$ 150 and 325	20 (25.6%)
More than US$ 325	28 (35.9%)

### Methodological procedures

The work was carried out between the months of September 2011 and February 2012. Visits were paid monthly during the day to the homes of wild bird keepers in the urban part of the city of Santana dos Garrotes, state of Paraíba. All streets of the city were surveyed to identify homes where wild birds were kept as pets. Attempts were made to establish an amicable relation with the wild bird keepers so that they would participate in the research, since they were aware that keeping wild animals without authorization is illegal. Nonetheless, in some cases, the residents refused (n = 35) to provide information about the subject researched. Before each interview, the nature and objective of the research were explained, and permission from the interviewees was requested to record the information. After the first interviews, other participants were selected by the snow ball technique [60], which consists in locating other potential research interviewees based on the indications of the first ones.

In total, 78 wild bird keepers (23 women and 55 men) agreed to participate in the study. The data were collected by means of semi-structured questionnaires, free interviews and informal conversations [61]. The questionnaires contained questions on the name of the bird, reason for its keeping, and manner of acquisition and maintenance. Information relative to the quantity of specimens kept and conditions for maintenance and feeding of the birds were obtained through direct observations in the homes. The birds were photographed, and the names of the animals were recorded as mentioned by the interviewees. The classification and nomenclature of the species recorded are in accordance with the Brazilian Commitee of Ornithological Registrations [36]. The birds recorded were identified at the species level with the help of field guides [62,63], through direct visualization and photographic records during the interviews. The ethical approval for the study was obtained from the Ethics committee of Paraiba University State (N° of protocol: 0146.0.126.000-10).

### Data analysis

An accumulation curve of the bird species cited by interviewees and recorded in the homes visited was prepared. In an accumulation curve for ethnobiological data, the X-axis corresponds to the number of individuals interviewed and Y-axis the number of species captured or utilized by the individuals for some purpose. The curve was randomized 1000 times and the means were calculated using the software EstimateS© version 8.2 [64]. EstimateS© permits the statistical analysis of species richness (for this work, species richness can be interpreted as the richness of species locally exploited) of samples by determination of the Chao2 index [65]. This index has been utilized in ethnozoological studies [66-68].

The non-parametric estimator Chao2 [69] is calculated by the following formula (a):

(a) $$\mathrm{Chao}2\;=\;\mathrm{Sobs}\;+\;\left(\frac{L^{2}}{2M}\right)$$

where: Sobs corresponds to the number of species in a given sample, L is the number of species in only one sample (“uniques”), and M is the number of species that occur exactly in two samples. The utilization of the Chao2 estimator is recommended for ethnozoological studies since it is a non-parametric estimator based on data of incidence.

The data were entered in EstimateS© using a spreadsheet of type of respondent (rows) x type of species (columns). In preparing the spreadsheet, a value of 1 was given for each species mentioned by an interviewee and 0 for those that were not recorded.

Descriptive statistics were utilized to assess the influence of socioeconomic aspects (income, sex, and age - see Table 1) on the practice of rearing wild birds as pets. The Spearman rank-order correlation was used to determine the existence of a relation between age of the interviewees and the number of species raised as pets. The Mann–Whitney *U* test was utilized to compare the number of species kept in captivity and the sex of the interviewee. The Kruskal-Wallis H test was employed for comparison between the number of species kept as pets by individuals belonging to three established income levels (1- less than R$ 300, 2 - R$ 300 to R$ 650, and 3 –greater than R$ 650). All statístical tests were done with the help of the STATISTICA version 10 program [70] and the level of significance was 5% in all cases.

## Results and discussion

### Species exploited

Through interviews and home visits with 78 wild bird keepers, 521 birds were counted, corresponding to 13 families, 30 genera and 40 species (Table 2). All species recorded are native to Brazil, of which 3 are endemic to the Caatinga (*Aratinga cactorum*, *Paroaria dominicana* and *Sporophila albogularis*), two endemic to Brazil (*Icterus jamacaii* and *Cyanocorax cyanopogon*) and only one species (*Sporagra yarrellii*) figures in Brazil’s official list of endangered species [22] and is categorized as “vulnerable” on the Red List of the IUCN [71].

**Table 2:** Bird species kept by bird-keepers interviewed (n = 78) in the city of Santana dos Garrotes, Paraíba, Brazil, including the number of specimens (N) and use by local people

**TAXA**	**Common name**	**N**	Use*
	**[Portuguese]**		
TINAMIDAE Gray, 1840			
*Nothura boraquira* (Spix, 1825)	White-bellied Nothura	10	(P),(C),(F)
	[Cordiniz]		
ANATIDAE Leach, 1820			
*Dendrocygna viduata* (Linnaeus, 1766)	White-faced Whistling-Duck	16	(P),(C),(F)
	[Marreca]		
*Amazonetta brasiliensis* (Gmelin, 1789)	Brazilian Teal	03	(P)
	[Paturí]		
CARIAMIDAE Bonaparte, 1850			
*Cariama cristata* (Linnaeus, 1766)	Red-legged Seriema	02	(P)
	[Seriema]		
COLUMBIDAE Leach, 1820			
*Columbina minuta* (Linnaeus, 1766)	Plain-breasted Ground Dove	03	(P)
	[Rolinha-cafifa]		
*Columbina talpacoti* (Temminck, 1811)	Ruddy Ground Dove	14	(P),(F)
	[Rolinha-caldo-de-feijão]		
*Columbina squammata* (Lesson, 1831)	Scaled Dove	14	(P)
	[Rolinha-cascavel]		
*Columbina picui* (Temminck, 1813)	Picui Ground Dove	07	(P)
	[Rolinha-branca]		
*Patagioenas picazuro* (Temminck, 1813)	Picazuro Pigeon	02	(P)
	[Asa-branca]		
*Zenaida auriculata* (Des Murs, 1847)	Eared Dove	12	(P),(C),(F)
	[Ribaçã]		
*Leptotila verreauxi* Bonaparte, 1855	White-tipped Dove	06	(P)
	[Juriti]		
PSITTACIDAE Rafinesque, 1815			
*Aratinga cactorum* (Kuhl, 1820)****	Cactus Parakeet	49	(P),(C)
	[Ganguirro]		
*Forpus xanthopterygius* (Spix, 1824)	Blue-winged Parrotlet	13	(P)
	[Papacú]		
*Amazona aestiva* (Linnaeus, 1758)	Turquoise-fronted Parrot	13	(P),(C)
	[Papagaio]		
CORVIDAE Leach, 1820			
*Cyanocorax cyanopogon* (Wied, 1821)***	White-naped Jay	07	(P),(C)
	[Cancão]		
TURDIDAE Rafinesque, 1815			
*Turdus rufiventris* Vieillot, 1818	Rufous-bellied Thrush	06	(P)
	[Sabiá-laranja]		
*Turdus amaurochalinus* Cabanis, 1850	Creamy-bellied Thrush	03	(P)
	[Sabiá-branca]		
COEREBIDAE d’Orbigny & Lafresnaye, 1838			
*Coereba flaveola* (Linnaeus, 1758)	Bananaquit	01	(P)
	[Sibito]		
THRAUPIDAE Cabanis, 1847			
*Saltator similis* d’Orbigny & Lafresnaye, 1837	Green-winged Saltator	02	(P)
	[Trinca-ferro]		
*Lanio pileatus* (Wied, 1821)	Pileated Finch	05	(P)
	[Maria-fita]		
*Tangara sayaca* (Linnaeus, 1766)	Sayaca Tanager	05	(P)
	[Azulão-de-rua]		
*Paroaria dominicana* (Linnaeus, 1758)****	Red-cowled Cardinal	79	(P),(C)
	[Galo-de-Campina]		
EMBERIZIDAE Vigors, 1825			
*Zonotrichia capensis* (Statius Muller, 1776)	Rufous-collared Sparrow	05	(P),(C)
	[Capa-bode]		
*Sicalis flaveola* (Linnaeus, 1766)	Saffron Finch	29	(P),(C)
	[Canário-da-terra]		
*Sicalis luteola* (Sparrman, 1789)	Grassland Yellow-Finch	04	(P)
	[Canário-de-lote]		
*Volatinia jacarina* (Linnaeus, 1766)	Blue-black Grassquit	04	(P),(C)
	[Tizil]		
*Sporophila lineola* (Linnaeus, 1758)	Linned Seedeater	36	(P),(C)
	[Bigodinho]		
*Sporophila nigricollis* (Vieillot, 1823)	Yellow-bellied Seedeater	27	(P),(C)
	[Mistriz]		
*Sporophila albogularis* (Spix, 1825)****	White-throated Seedeater	67	(P),(C)
	[Golado]		
*Sporophila leucoptera* (Vieillot, 1817)	White-bellied Seedeater	03	(P)
	[Chorão]		
*Sporophila bouvreuil* (Statius Muller, 1776)	Cooper Seedeater	10	(P),(C)
	[Caboclinho]		
CARDINALIDAE Ridgway, 1901			
*Cyanoloxia brissonii* (Lichtenstein, 1823)	Ultramarine Grosbeak	06	(P)
	[Azulão-da-mata]		
ICTERIDAE Vigors, 1825			
*Procacicus solitarius* (Vieillot, 1816)	Solitary Black Cacique	03	(P)
	[Bico-de-osso]		
*Icterus pyrrhopterus* (Vieillot, 1819)	Variable Oriole	14	(P),(C)
	[Xexeu]		
*Icterus jamacaii* (Gmelin, 1788)***	Campo Troupial	14	(P),(C)
	[Chofreu]		
*Gnorimopsar chopi* (Vieillot, 1819)	Chopi Blackbird	07	(P),(C)
	[Craum]		
*Chrysomus ruficapillus* (Vieillot, 1819)	Chestnut-capped Blackbird	05	(P),(C)
	[Pardal-do-papo-roxo]		
*Molothrus bonariensis* (Gmelin, 1789)	Shiny Cowbird	12	(P),(C)
	[Pardal-preto]		
FRINGILLIDAE Leach, 1820			(P)
*Sporagra yarrellii* (Audubon, 1839)**	Yellow-faced Siskin	02	(P)
	[Pintasilgo]		
*Euphonia chlorotica* (Linnaeus, 1766)	Purple-throated Euphonia	01	(P)
	[Vivim]		
**TOTAL**		521	

*Use: (P) Pets; (F) Food; (C) Commercial, **Endangered (Brazil) and Vulnerable (IUCN), ***Endemic to Brazil, ****Endemic to the Caatinga.

Most of the birds (90%) were pets, while 10% of the wild species were raised for food. This situation is in accordance with Albuquerque et al. [46], who pointed out that the main reason for the high frequency of wild birds hunted in the Caatinga is tied mainly to their use as pets, which can be considered the main stimulus for the illegal sale of birds in the region. Without a doubt, in various Northeast cities, there are public stores and open markets where wild birds are illegally sold for the purpose of pets [9,10,43,44,72,73].

**Figure 2:**
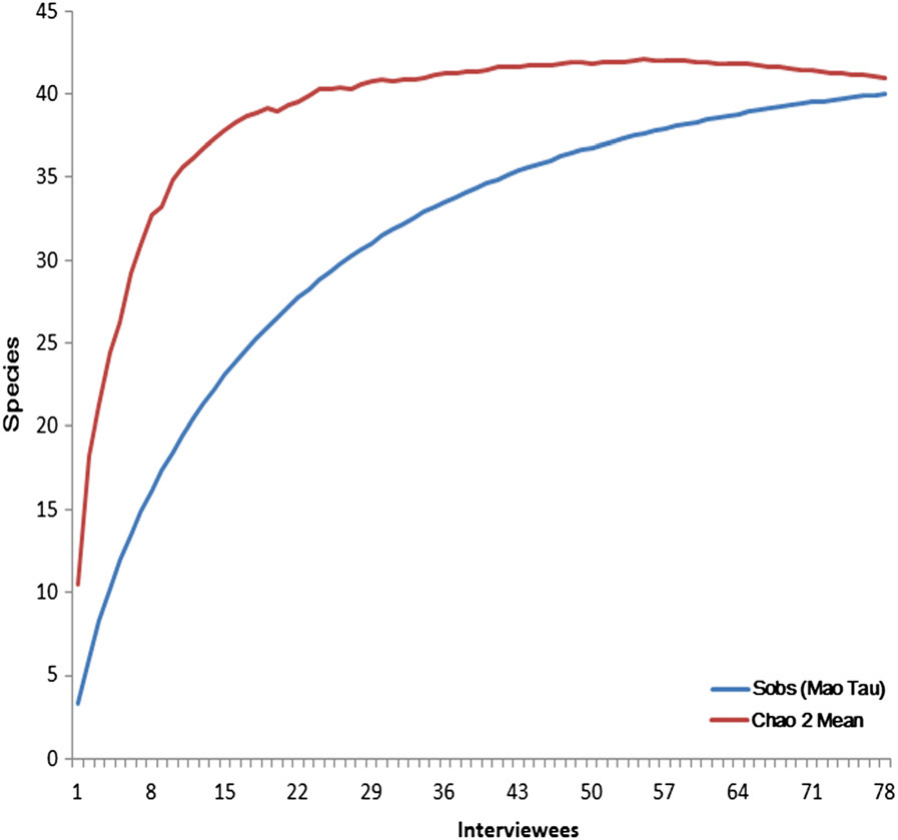
**Graphs showing the values obtained with the richness estimators of bird species kept as pets (based on data from 78 bird-keepers) in the city of Santana dos Garrotes (Paraíba State, Northeast Brazil).** Number of Species Observed (Sobs = 40), Number of species estimated (Chao2 = 40.99 ± 1.44).

Based on the data collected, the mean number of species observed (Sobs) was compared with that expected to be kept in the surveyed area (Table 2, Figure 2). The results demonstrated the sampling efficiency was adequate, since 97.6% (n = 40) of wild species kept as pets in the region investigated (n1 ≈ 41, Chao2 = 40.99 ± 1.44) were recorded. The species accumulation curve showed a tendency to stabilize. These results provided evidence that ethno-ornithological studies constitute a tool for the rapid understanding of the interactions established between local inhabitants and the wild avifauna. In particular, it is evident that richness estimators are useful in determining the success of data collection, since many inhabitants refused to participate in this type of study. The reasons were almost always connected to the fear of some type of legal action, since the capture, persecution/apprehension or slaughter of wild animals is against the law in the majority of communities in Brazil (Federal Law No. 5.197 of January 3, 1967). Alves and Souto [74], for example, noted that this type of problem is frequent in ethnozoological studies in Brazil.

The families with largest number of species recorded were in Emberizidae (n = 9 species), followed by Columbidae (n = 7), Icteridae (n = 6) and Psittacidae (n = 3). This distribution coincides with other studies related to the use and sale of wild birds [10,28], which recorded that birds belonging to these families are often captured and utilized by the people who live in the semi-arid areas [5,9,28,50,75] and other regions of Brazil [10]. For example, Fernandes-Ferreira et al. [53], reported that emberizids and icterids compose 40% of the wild birds raised and sold in the countryside of Ceará. In markets of the city of Campina Grande, Rocha et al. [44] observed that 48% of the total birds belonged to the Emberizidae, demonstrating the evident popular preference for this family in relation to other groups of songbirds. In a recent study, Alves et al. [28] reported the families Emberizidae and Columbidae as the most frequent among the birds used as pets in Catolé do Rocha, a municipality of the Paraíba semi-arid region.

**Figure 3:**
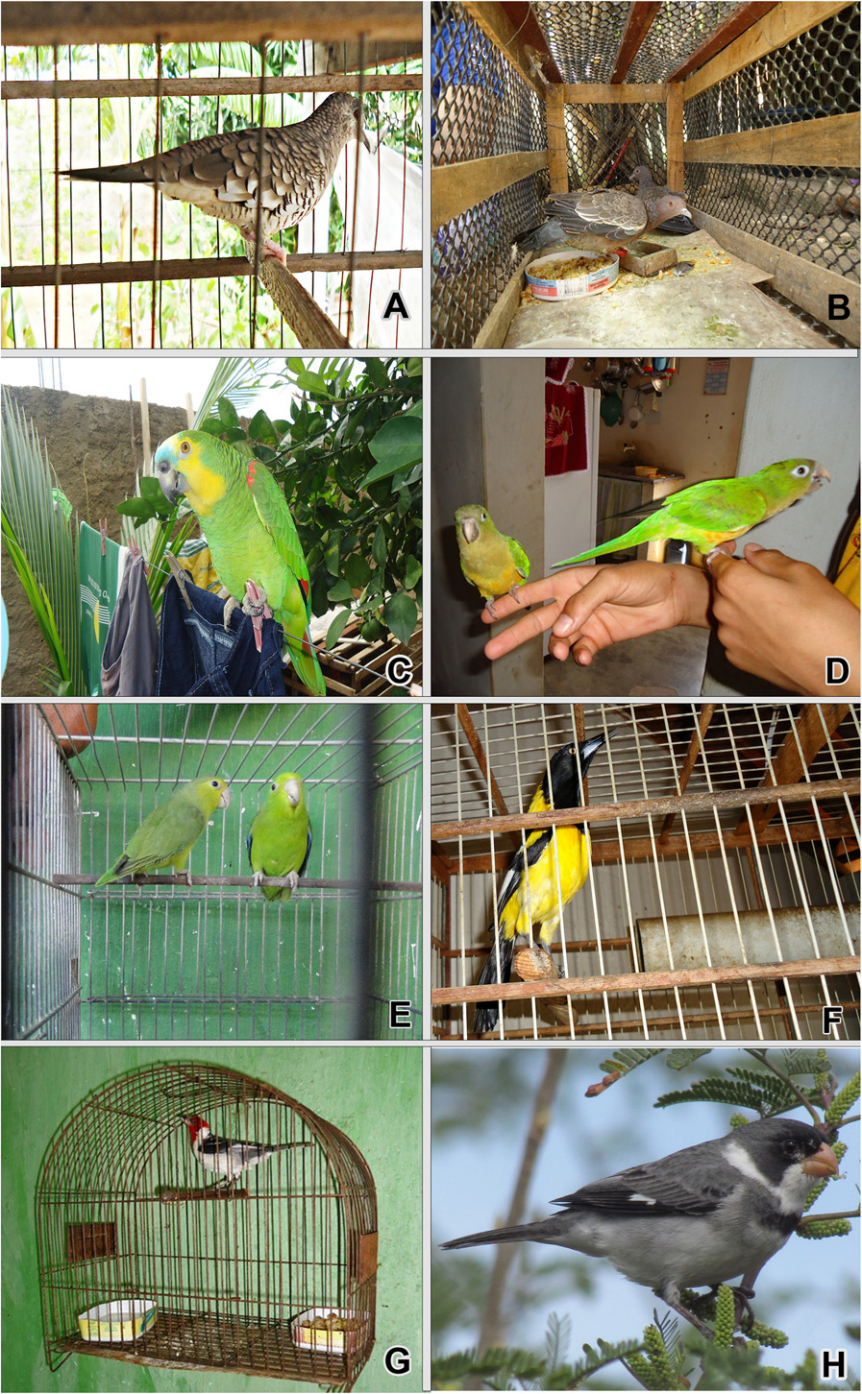
**Examples of species kept in captivity in city of Santana dos Garrotes (Paraíba State, Northeast Brazil). A** - *Columbina squammata*; **B** - *Patagioenas picazuro*; **C** - *Amazona aestiva*; **D** - *Aratinga cactorum*, **E** - *Forpus xanthopterygius*; **F** - *Icterus jamacaii*; **H** - *Paroaria dominicana,* and **D** - *Sporophila albogularis.*

The birds that were most often recorded were *Paroaria dominicana* (n = 79 specimens), *Sporophila albogularis* (n = 67 specimens), *Aratinga cactorum* (n = 49 specimens), *Sporophila lineola* (n = 36 specimens), *Sicalis flaveola* (n = 29 specimens) and *Sporophila nigricollis* (n = 27 specimens) (Figure 3). The red-cowled cardinal (*Paroaria dominicana*), the most recorded species, is one of the most common pet birds in Northeast Brazil [5,9,28,50]. Sick [47], mentioned their predilection in connection with the illegal sale of wild birds. The white-throated seedeater (*Sporophila albogularis*) and cactus parakeet *(Aratinga cactorum)* are also very popular. The last, like the majority of psittacids, is often caught because of its charm, particularly docility, beauty and its ability to imitate sounds, including human voices [48]. The popularity of these species has been reported in various studies on the sale of birds in various cities of Brazil [44,72,76,77].

Despite the legal prohibitions, the high number of species of wild birds used as pets is not surprising, considering that birds are often utilized for these purposes in Brazil [10] and such uses have occurred in a clandestine or semi-clandestine manner. Our results corroborated a tendency shown by other studies that point to the cultural importance of the hobby of raising birds as pets, a practice that has been perpetuated in the country [9,10,72,78]. Examples of species kept in captivity are indicated in Figure 3.

Statistical analyses showed that there was no significant correlation (p > 0.05) between the number of species of birds kept as pets and the age of the bird keepers. Similarly, the number of species of birds kept as pets was not influenced by the sex (Mann–Whitney *U* test = 473.5, p > 0.05) or income of these keepers (Kruskal-Wallis test H = 1.80, d.f. = 2, n = 78, p = 0.4). The lack of correlation found between socioeconomic factors and keeping birds as pets reflects the cultural importance of this practice of rearing wild birds for pets in the region, which is widespread among the local population, independent of socioeconomic factors.

### Maintenance of birds

**Figure 4:**
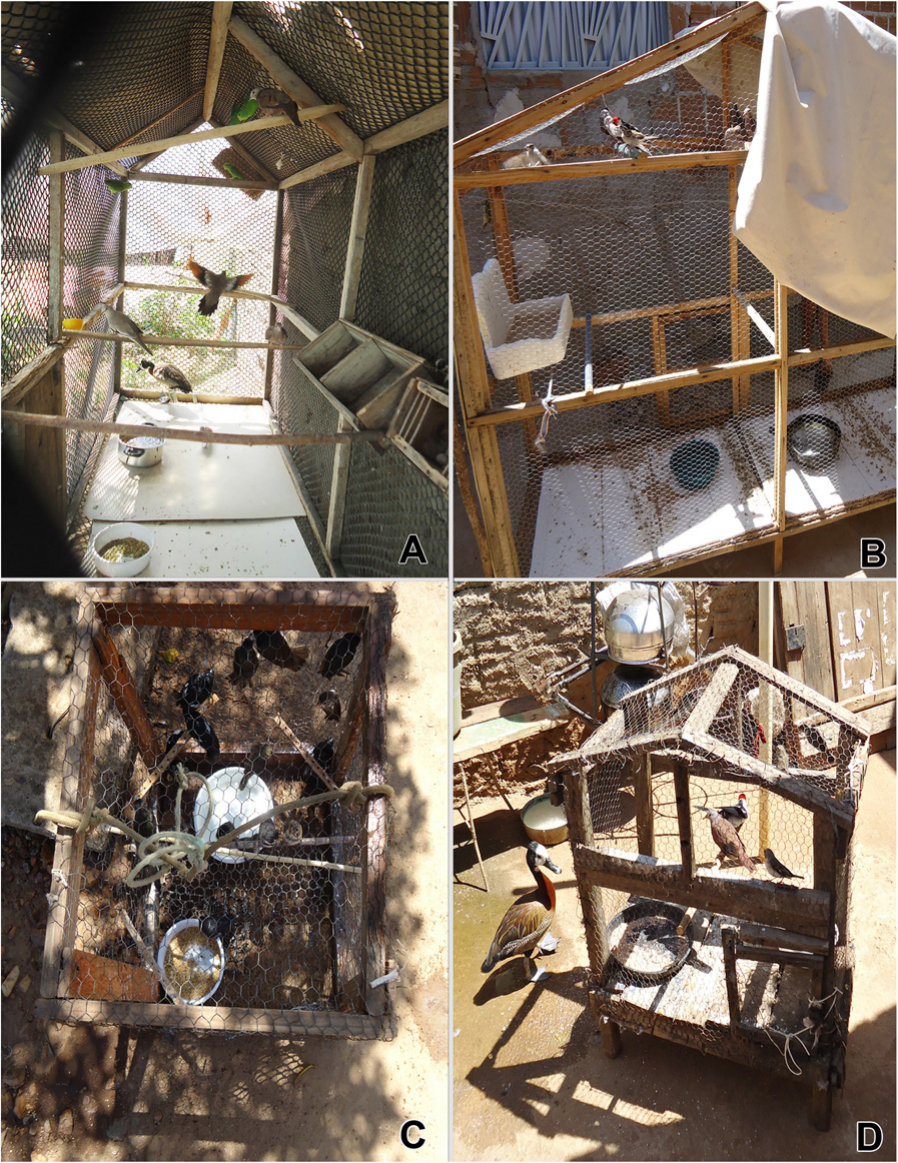
Aviaries in which wild birds are kept in our study area.

The majority of the species recorded in the area studied were kept in cages or aviaries. The cages were hung from the ceiling of the houses or placed on stands, which generally use to house one bird. When there was more than one specimen in the same cage, they were usually couples or small groups belonging to the same species. However, in the aviaries, depending on the size, a large number of birds could be kept together, regardless of the number of different species (Figure 4). The aviaries are large enclosures (compared to cages) and stationary, in which a large number of specimens are kept. These are made of masonry, screen or wire grid. Some birds recorded were raised loose, flying around freely inside the bird keeper’s home or backyard, although depending on food furnished by the bird keepers.

Cleaning of the enclosure where the bird was kept was done daily, or two or three times a week. When asked about the nutrition of the birds, the bird keepers mentioned various foods that were offered according to the preference of the species. Seeds, fruits and human food were the common items in the birds’ diet. Some species had a more restricted diet, while others fed on various foods. Food was offered every day or on alternating days or replaced when needed. Birds that fed on fruits needed their food replaced daily. Besides feeding, the wild bird keepers (n = 37) supplied vitamin supplements to keep the birds always healthy, singing and with a good appearance.

Although the dimensions of the cages were not noted, it was apparent that some cages were clearly too small to house one or more specimens, which made it difficult for them to fly around in these enclosures. Previous studies have demonstrated that inadequate conditions for keeping birds can cause death or complications with their health [43,44]. For example, small cages can cause atrophy of the muscular system of birds and pain [79], affecting even their behavior, besides favoring the transmission of zoonosis.

Among the interviewees, 75.6% (n = 59) stated that the birds maintained in captivity were exposed to various diseases, and 24.4% (n = 19) said that they did not know when the bird were sick. Among those that recognized sick birds, 37 responded that they treated the diseases, 17 reported that they released the birds for them to seek cures in nature, and 5 mentioned that they did not treat the birds. Treatment included the use of vitamins especially for birds and offered chicken eggs, both prescribed for strengthening sick birds. The bird keepers also used medications such as sodium dipyrone. Still, according to the interviewees, feeding of the birds should be controlled, since depending on the type and quantity of food offered, it can result in eating disorders leading to death. Licarião [80] reported similar precautions for wild birds in the municipality of Campina Grande, Paraíba. According to this author, precautions can be taken by the wild bird keepers, thereby independent of any consult with a specialist. Generally, bird keepers are familiar with such aspects through the exchange of information with other bird keepers or parents or from their own experience.

### Conservationist implications

The Caatinga is the Brazilian domain whose biodiversity is least known. The avifauna is included in this context, which has suffered a series of threats, some of them to a particular species or locality. For example, we can cite the impact caused by activities of mineral extraction or disorganized tourism [81]. However, it is known that some threats are common to practically all the biome, where the principal ones are habitat loss, caused mostly by deforesting, and the capture of birds, whether for food or for breeding animals or even for illegal sale [9,10,28,81]. Despite the clear influence of human activities on the avifauna of the region, studies on the interactions between humans and the birds in the Caatinga have emerged only recently, although such information is essential for defining conservation strategies.

Despite the well-known legal implications and eventual confiscation of the wild birds, as well as the arrest of people who breed or trade these animals, these activities persist and flourish in a socioeconomic and cultural manner since current laws are known to be inefficient [10,27]. The data obtained in the present work corroborates this reality, which is common in all the semi-arid northeastern region, where hunting is widespread, whether for cultural or economic reasons [9,28,50,56,75,82].

Bird-keeping is a culturally deep-rooted practice, where it is common not only in small cities or villages in the countryside, as observed in the area studied, but also in large urban centers [9,10,81], which has culminated in the persistent illegal trade of birds in the Caatinga, especially in the lowland area, where the predominance of an arbustive vegetation [83], and large number of roads and paths favor the capture of birds, which are sold in public shops and street markets of various cities [10,46], or even among the wild bird keepers themselves, as a way of circumventing laws that prohibit such activities. Alves et al. [10] points out that the capture and sale of birds involve many players, forming a large commercial network responsible for their distribution to different regions.

Trade is a serious threat to the conservation of various species of birds in Brazil [10,23,48]. While some species are destined for exportation, the heart of the bird market is to meet local demand. Estimates indicate that approximately 4 million birds [84] are traded illegally in the country, and of these, 70% are destined for national sales and the rest for export [42]. A review recently published by Alves et al. [10] revealed that at least 295 different species of birds are sold illegally in Brazil as pets, with estimates pointing to 400 species or more – approximately 23% of the total number of species of native birds of Brazil. In the majority of Brazilian cities, there are bird markets, and unfortunately, very little is done to regulate and monitor sales to guarantee their legality and sustainability [48]. The majority of the specimens are sold as pets, while some are sold for food and, on a smaller scale, for medicinal and magical-religious purposes [14,56,67,85-90].

The capture of wild birds for keeping in captivity, prompted by their song or the beauty of their plumage, is one of the main causes of population decline of various species [19,47,91,92]. One example of a wildlife species that is currently being unsustainably poached is the parrot (Family: Psittacidae) [93,94]. The Hyacinth Macaw *Anodorhynchus hyacinthinus*, for example, is mainly threatened by a large and persistent illegal trade. At least 10,000 of these birds were taken from the wild in the 1980s, and 50% of them were absorbed by the Brazilian market [95]. Similarly, Golden Parakeet *Guaruba guarouba* is trapped for trade and is highly sought after by both international and national markets. There is a well-organized internal trade of Red-spectacled Amazon parrots *Amazona pretrei*, and these birds are usually taken by cutting down their nest-tree, resulting in the permanent abandonment of that nesting site. Many other parrot species may likewise become threatened if illegal trading is allowed to continue [10]. Aside from the question of legality, the clandestine capture of wild birds generates a series of serious environmental consequences. The removal of wild birds from nature can lead, in the medium- and long-term, to species extinctions [96], and compromise several ecological services, such as pollination, seed dispersal, and control of populations of other animals [47,97-99].

The National Action Plan for the conservation of birds of the Caatinga threatened by extinction (PAN birds of the Caatinga) [81] indicates 12 priority species for conservation in the biome, since these occupy some type of threat status [40,71]. In relation to these species as principal threats are habitat loss and hunting. The same situation is applies to others, which even though not on lists of threatened species are widely utilized as pets, as observed in the area studied and in other localities of the semi-arid Northeast [5,28,50,75].

Obviously, there are economic and cultural questions when considering the hunting of wild animals in the semi-arid Northeast [9,27,100]. In the case of birds, many species are locally utilized as a source of food or kept as pets [10,38,47,101]. However, the use of animals is often limited to the family unit or to small groups of people and has gained prominence in everyday business [10,72,81,102].

In view of the widespread use and illegal sale of wild birds in Brazil and its implications for conservation, there is an urgent need now for the implementation of measures aimed at controlling these activities, which should consider cultural, economic, social and ecological aspects. These measures should focus on the direct fight against the illegal trafficking of animals as well as educational campaigns that reach all the players involved, from the collector to the consumer/keeper.

In Brazil, a variety of wild vertebrate species are kept as ‘pets’ including fishes, amphibians, reptiles, birds and mammals [103-107]. Although studies on the use and sale of these animal groups as pets are scarce, the information available indicates that birds are the principal taxon exploited for this purpose and that they have endured the greatest impact, particularly considering illegal trade [10].

The current situation with the exploitation of the wild avifauna in the semi-arid Northeast demonstrates that conservation measures should be implemented mainly through public policies [9,10,108,109]. First, it is necessary to implement outreach and education programs about the environmental consequences that result from the trade of these animals. In essence, the wild bird keepers do this by their admiration for the birds, which can be utilized to raise their conscience about this activity. Reducing demand consequently decreases the capture of the birds [53]. Concomitantly, there is an urgent need for projects aimed at promoting the protection and recovery of ecosystems [9,53], since the greatest threat to birds of the Caatinga is habitat loss. The creation of conservation units in this biome can be encouraged to mitigate this threat. For already existing conservation units of federal, state and municipal jurisdiction, there is a need for a greater supervision on the part of responsible agencies to resolve problems of degradation and non-sustainable exploitation of plant and animal resources.

The great pressure by humans on the avifauna of the Caatinga indicates that conservation measures should incorporate the interactions between the people and birds of the region and their social dimensions, and therefore, ethno-ornithological studies are essential because they can provide basic information for designing urgent conservation strategies, as well as promoting public policies capable of easing the current situation with the over-exploitation of birds in the regional sense.

## Competing interests

The authors declare that they have no competing interests.

## Authors’ contributions

RRNA, RCLL, WMSS and ALR – Writing of the manuscript, literature survey and interpretation, and analysis of taxonomic aspects; RCLL –Ethnozoological data. All authors read and approved the final manuscript.
